# MicroRNA-184 is a downstream effector of albuminuria driving renal fibrosis in rats with diabetic nephropathy

**DOI:** 10.1007/s00125-017-4248-9

**Published:** 2017-03-31

**Authors:** Cristina Zanchi, Daniela Macconi, Piera Trionfini, Susanna Tomasoni, Daniela Rottoli, Monica Locatelli, Michael Rudnicki, Jo Vandesompele, Pieter Mestdagh, Giuseppe Remuzzi, Ariela Benigni, Carlamaria Zoja

**Affiliations:** 1 IRCCS – Istituto di Ricerche Farmacologiche Mario Negri, Centro Anna Maria Astori, Science and Technology Park Kilometro Rosso, Via Stezzano 87, 24126 Bergamo, Italy; 2 Medical University Innsbruck, Department of Internal Medicine IV–Nephrology and Hypertension, Innsbruck, Austria; 3 Biogazelle, Zwijnaarde, Belgium; 4 Unit of Nephrology and Dialysis, Azienda Socio Sanitaria Territoriale (ASST) Papa Giovanni XXIII, Bergamo, Italy; 5 Department of Biomedical and Clinical Sciences, University of Milan, Milan, Italy

**Keywords:** Albuminuria, Diabetic nephropathy, Fibrosis, miR-184, Zucker diabetic fatty rats

## Abstract

**Aims/hypothesis:**

Renal fibrosis is a common complication of diabetic nephropathy and is a major cause of end-stage renal disease. Despite the suggested link between renal fibrosis and microRNA (miRNA) dysregulation in diabetic nephropathy, the identification of the specific miRNAs involved is still incomplete. The aim of this study was to investigate miRNA profiles in the diabetic kidney and to identify potential downstream targets implicated in renal fibrosis.

**Methods:**

miRNA expression profiling was investigated in the kidneys of 8-month-old Zucker diabetic fatty (ZDF) rats during overt nephropathy. Localisation of the most upregulated miRNA was established by in situ hybridisation. The candidate miRNA target was identified by in silico analysis and its expression documented in the diabetic kidney associated with fibrotic markers. Cultured tubule cells served to assess which of the profibrogenic stimuli acted as a trigger for the overexpressed miRNA, and to investigate underlying epigenetic mechanisms.

**Results:**

In ZDF rats, miR-184 showed the strongest differential upregulation compared with lean rats (18-fold). Tubular localisation of miR-184 was associated with reduced expression of lipid phosphate phosphatase 3 (LPP3) and collagen accumulation. Transfection of NRK-52E cells with miR-184 mimic reduced LPP3, promoting a profibrotic phenotype. Albumin was a major trigger of miR-184 expression. Anti-miR-184 counteracted albumin-induced LPP3 downregulation and overexpression of plasminogen activator inhibitor-1. In ZDF rats, ACE-inhibitor treatment limited albuminuria and reduced miR-184, with tubular LPP3 preservation and tubulointerstitial fibrosis amelioration. Albumin-induced miR-184 expression in tubule cells was epigenetically regulated through DNA demethylation and histone lysine acetylation and was accompanied by binding of NF-κB p65 subunit to miR-184 promoter.

**Conclusions/interpretation:**

These results suggest that miR-184 may act as a downstream effector of albuminuria through LPP3 to promote tubulointerstitial fibrosis, and offer the rationale to investigate whether targeting miR-184 in association with albuminuria-lowering drugs may be a new strategy to achieve fully anti-fibrotic effects in diabetic nephropathy.

**Electronic supplementary material:**

The online version of this article (doi:10.1007/s00125-017-4248-9) contains peer-reviewed but unedited supplementary material, which is available to authorised users.

## Introduction

Diabetic nephropathy is one of the major microvascular complications of diabetes and the leading cause of chronic kidney disease and end-stage renal disease (ESRD) throughout the world [1, 2]. A typical hallmark of diabetic nephropathy is excessive deposition of extracellular matrix (ECM) proteins in the mesangium and tubulointerstitium, culminating in glomerulosclerosis, interstitial fibrosis, tubular atrophy and loss of renal function [3–5]. Other key features include interstitial accumulation of inflammatory leucocytes and matrix-producing myofibroblasts [6, 7]. Fibrosis is at the core of the high morbidity and mortality rates associated with diabetic nephropathy but specific therapeutic options with this target are not yet available in clinics. Studies in animal models of diabetes have contributed to defining intracellular and molecular pathways driving renal fibrosis, which include the activation of the renin–angiotensin system, protein kinase C, TGF-β1 and monocyte chemoattractant protein-1 (MCP-1) and the upregulation of plasminogen activator inhibitor-1 (PAI-1), connective tissue growth factor (CTGF)/CCN2, collagen and cytokines [8–11]. Recent studies link fibrosis to changes in microRNAs (miRNAs), a class of short (21–24 nucleotides) noncoding RNAs that regulate gene expression through post-translational and epigenetic mechanisms and thereby affect several cellular processes, from development to disease conditions [12–14]. A number of miRNAs have been shown to be relevant to fibrotic processes in diabetic nephropathy, including miR-29 and miR-200 families, miR-192 and miR-21 [14–17]. These miRNAs are regulated by TGF-β in renal cells, and normalisation of their expression ameliorated fibrosis in in vitro and in vivo models of diabetes, suggesting that targeting these miRNAs could be a way to improve diabetic nephropathy downstream of TGF-β [16]. The in vivo findings were mainly obtained in early stages of type 1 and type 2 diabetic nephropathy [14, 16].

The objective of our study was to use a rat model of type 2 diabetes (Zucker diabetic fatty [ZDF] rats) to investigate miRNA profiles in the kidneys [18, 19] at an advanced phase of the disease, and to identify potential downstream targets implicated in renal fibrosis.

## Methods

### Experimental animals

Fifteen male ZDF (ZDF/Gmi-*fa/fa*) and ten non-diabetic lean (ZDF/Gmi-*fa/+*) rats were purchased from Charles River Laboratories Italia (Calco, Italy). For the first series of experiments, two groups of ZDF and lean rats (*n* = 5/group) at 8 months of age, after blood glucose and albuminuria measurement, were euthanised through CO_2_ inhalation and their kidneys were collected for morphological analyses, miRNA profiling, in situ hybridisation and immunohistochemistry. In a subsequent study, additional ZDF rats were randomised to receive the ACE inhibitor ramipril (1 mg/kg in the drinking water) or vehicle (water) (*n* = 5/group) from 4 to 8 months of age. Five lean rats served as controls. When rats were killed, albuminuria and creatinine clearance were determined and kidneys were processed for evaluation of miR-184 and *Pai-1* (also known as *Serpine1*) mRNA and for immunohistochemistry. The experimenters were not blind to the treatment, but they were blind for measurement of experimental outcomes. Rats were housed in a specific pathogen-free facility with constant temperature and a 12 h light–dark cycle. All procedures were carried out in compliance with national (D.L.n.26, March 4, 2014) and international laws and policies (directive 2010/63/EU) and were approved by the Institutional Animal Care and Use Committees of Mario Negri Institute (see ESM Methods for further details).

### MicroRNA expression profiling

The miRNA profile was generated from RNA isolated from frozen kidneys using the TaqMan Array Rodent miRNA cards (Life Technologies, Carlsbad, CA, USA). See ESM Methods.

### Quantitative RT-PCR

Quantitative RT (qRT)-PCR analyses of miR-184, *Lpp3* (also known as *Plpp3*) mRNA and *Pai-1* mRNA were performed in RNA isolated from kidney tissue using specific TaqMan assays (Life Technologies). See ESM Methods.

### In situ hybridisation and immunohistochemistry

miR-184 hybridisation was assessed on paraffin-embedded kidney sections using the double-digoxigenin-labelled LNA miRCURY probe (Exiqon, Vedbaek, Denmark). Staining for tubule markers (aquaporin1 and Tamm–Horsfall protein), lipid phosphate phosphatase 3 (LPP3) and type III collagen was performed on kidney serial sections by immunoperoxidase using the following rabbit primary antibodies: anti-aquaporin1 (1:100; Santa Cruz Biotechnology, Santa Cruz, CA, USA), anti-Tamm–Horsfall protein (1:100; Santa Cruz Biotechnology), anti-LPP3 (1:200; Biorbyt, Cambridge, UK), anti-type III collagen (1:100; Chemicon, Temecula, CA, USA). Double labelling for LPP3 and α-smooth muscle actin (α-SMA) was assessed by immunofluorescence using rabbit anti-LPP3 antibody (1:100; Biorbyt) and Cy3-conjugated mouse anti-α-SMA antibody (1:200; Sigma-Aldrich, St. Louis, MO, USA) (see ESM Methods for further details).

### Bioinformatic analysis of miR-184 target genes

The microRNA body map web tool (www.mirnabodymap.org/), the miRanda-mirSVR (http://microRNA.org/) and the EIMMo miRNA target prediction software (http://mirz.unibas.ch/EIMMo3/) were used to identify potential targets of miR-184. Details are provided in ESM Methods.

### In vitro studies

#### Luciferase assay

Human AD-293 cells (Agilent, Santa Clara, CA, USA; negative for mycoplasma contamination) were co-transfected with a construct containing the human *LPP3*–3′ untranslated region (UTR) downstream of the Firefly luciferase gene, the co-reporter vector pRL-TK encoding the Renilla luciferase and rat miR-184 mimic or control mimic using Lipofectamine 2000 (Life Technologies). After 48 h, reporter activity was measured with the Dual-Luciferase Reporter (DLR) Assay System (Promega, Madison, WI, USA) (see ESM Methods for further details).

#### Cell transfection and incubation

Rat proximal tubule NRK-52E cells (DSMZ, Braunschweig, Germany, negative for mycoplasma contamination) [20] were transfected with miR-184 mimic or control mimic using Lipofectamine 2000. Cells were collected 24 and 48 h later for analyses of *Lpp3*, *Ctgf/Ccn2*, *Pai-1*, *Tgf-β* (also known as *Tgfb1*) and *Mcp-1* (*Ccl2*) mRNA expression, and 72 h later for western blot of LPP3. In selected experiments transfected cells were exposed to ICG-001 (Selleckchem, Munich, Germany), an inhibitor of T cell factor (TCF)/β-catenin transcription, for *Pai-1* mRNA analysis. To identify triggers of miR-184 expression, NRK-52E cells were exposed to angiotensin II, human serum albumin, holo-transferrin, TGF-β1 or fatty acid (FA)-free albumin (Sigma-Aldrich). NRK-52E cells were transfected with anti-miR-184 or miRNA inhibitor negative control before albumin stimulation for *Lpp3* and *Pai-1* mRNA evaluation. Epigenetic regulation of miR-184 was assessed in NRK-52E cells exposed to the chromatin-modifying drugs 5-aza-2′-deoxycytidine and 4-phenylbutyric acid for qRT-PCR of miR-184. Further, chromatin immunoprecipitation followed by quantitative (q)PCR was performed in albumin-treated NRK-52E cells. See ESM Methods.

#### qRT-PCR

RNA was isolated from NRK-52E cells for analysis of miR-184 expression and *Lpp3* and *Pai-1* mRNA using specific TaqMan assays. *Ctgf/Ccn2*, *Tgf-β* and *Mcp-1* mRNA were evaluated using SYBR Green and the primers listed in ESM Table 1 (see ESM Methods for further details).

#### Western blot analysis

NRK-52E cells were processed as previously described [20]. Immunodetection of LPP3 was performed using rabbit anti-LPP3 (1:300; Biorbyt). See ESM Methods.

#### Chromatin immunoprecipitation

Chromatin immunoprecipitation (ChIP) analysis was performed in albumin-treated NRK-52E cells using the following rabbit antibodies against: methylcytosine-binding protein 2 (MeCP2, 5 μg; ab2828, Abcam, Cambridge, UK), methyl-binding domain 1 (MBD1, 5 μg; sc-10751, Santa Cruz Biotechnology), acetylated histone H3 lysine 9 (H3K9ac, 2 μg; ab4441, Abcam), trimethylated histone H3 lysine 4 (H3K4me3, 2 μg; ab8580, Abcam) and NF-κB-p65 (5 μg; SC-372X, Santa Cruz Biotechnology) followed by qPCR using primers (ESM Table 1) in spanning genomic regions surrounding the miR-184 gene. See ESM Methods.

### Statistical analysis

Results are mean ± SEM. Data were analysed by ANOVA followed by the Tukey–Cicchetti test for multiple comparisons, or by Student’s *t* test for unpaired data, as appropriate. A *p* value <0.05 was considered statistically significant. Differentially expressed miRNAs were identified using R statistical software (https://cran.r-project.org/) by two non-parametric tests: the Mann–Whitney test and Rank Products algorithm (*p* < 0.05) and considering only the values with a fold change greater than 2.

## Results

### miR-184 is upregulated in the kidneys of ZDF rats

**Fig. 1:**
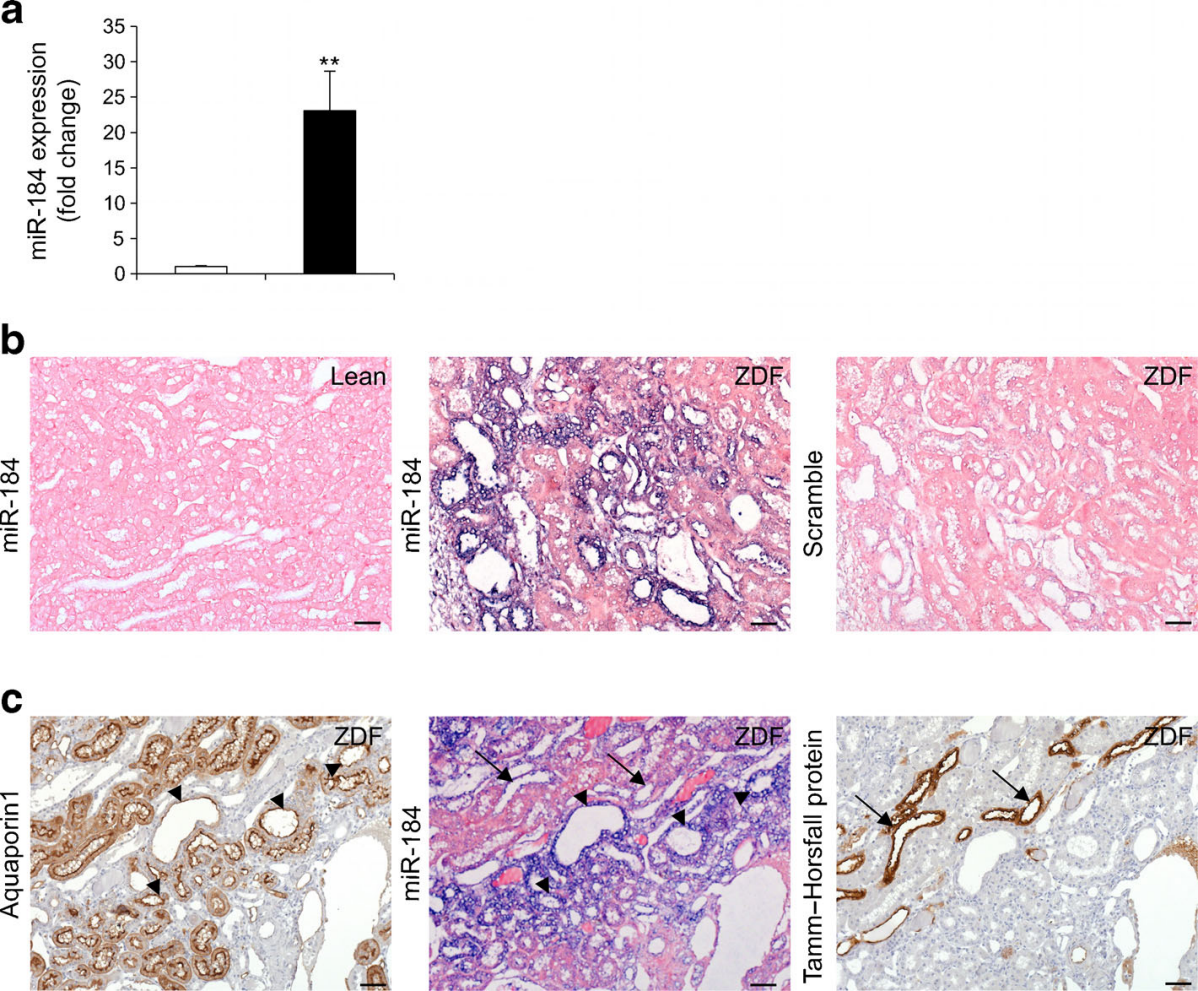
miR-184 is upregulated in the kidneys of ZDF rats. (**a**) miR-184 expression in lean (white bar) and ZDF (black bar) rats (*n* = 5/group). Data normalised to U87 small nucleolar RNA are reported as fold change relative to lean rats. **p < 0.01 vs lean rats. (**b**) Representative images of in situ hybridisation for miR-184 in kidney cortex from a lean rat, a ZDF rat and for scramble probe as negative control. (**c**) Staining for aquaporin1 (marker of proximal tubules), miR-184 and Tamm–Horsfall protein (marker of distal tubules) in serial kidney sections from a ZDF rat. Proximal (arrow heads) and distal (arrows) tubules positive for miR-184 are shown. Scale bars, 50 μm

Systemic and renal variables of the investigated rats are given in Table 1, and are in line with previous studies [18, 19]. MicroRNA expression profiling was performed in the kidneys of ZDF and lean rats at 8 months of age. Statistical analysis with two non-parametric tests (Mann–Whitney test and Rank Products algorithm) identified 15 miRNAs that were differentially expressed between ZDF and lean rats (ten upregulated and five downregulated) (ESM Table 2). miR-184 showed the strongest differential upregulation (18-fold). Data from Multiplex PCR were validated by qRT-PCR, and confirmed a 23-fold increase of miR-184 in ZDF rats (Fig. 1a), mainly localised at the tubular epithelium (Fig. 1b), in proximal and distal tubules, as revealed by aquaporin1 and Tamm–Horsfall protein staining (Fig. 1c). No signal for miR-184 was detected in the kidneys of lean rats (Fig. 1b). A weak expression was found in the glomeruli of ZDF rats (ESM Fig. 1a).

**Table 1:** Systemic and renal variables in ZDF and lean rats at 8 months of age

Group	Body weight (g)	Blood glucose (mmol/l)	Albuminuria (mg/day)	Glomerulosclerosis (%)	Tubular damage (score)
Lean rats	446 ± 7	5.84 ± 0.17	0.57 ± 0.10	0	0
ZDF rats	416 ± 6*	28.98 ± 0.99***	207 ± 28***	22 ± 5**	1.12 ± 0.1***

Values are mean ± SEM

*p < 0.05, **p < 0.01 and ***p < 0.001 vs lean rats

### LPP3 is a target of miR-184

**Fig. 2:**
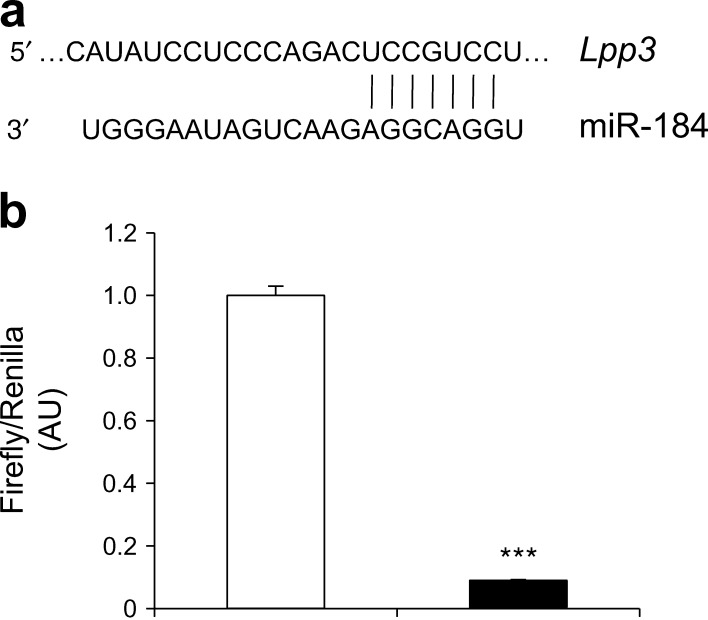
In vitro validation of *Lpp*3 as a target of miR-184*.* (**a**) Schematic representation of *Lpp3* 3′ UTR as a putative target for miR-184. The seed-recognising site (position 1272–1278 of rat *Lpp3* 3′ UTR) binding to miR-184 RNA is indicated. (**b**) Luciferase activity in AD-293 cells co-transfected with the reporter plasmid containing the *Lpp3* 3′ UTR downstream of the Firefly luciferase gene, the co-reporter vector pRL-TK encoding the Renilla luciferase and with control (white bar) or miR-184 (black bar) mimic. Firefly/Renilla luciferase activity was expressed as arbitrary units (AU). Normalised luciferase activity of control mimic-transfected cells was set to 1. ***p < 0.001 vs control mimic (*n* = 3 experiments)

To identify potential miR-184 target genes, miRNA target prediction algorithms miRanda and EIMMo were used in conjunction with the microRNA body map web tool. Of the putative candidates, we focused on *Lpp*3 because it was the only mRNA predicted as a target of miR-184 by all databases. Notably, the binding site for miR-184 in the 3′ UTR of *Lpp*3 (Fig. 2a) is evolutionarily conserved among species. To confirm that LPP3 is a true miR-184 target, we performed luciferase reporter assays using a plasmid carrying the full-length 3′ UTR of human *LPP3* downstream of the luciferase gene. Co-transfection of AD-293 cells with miR-184 mimic and the reporter plasmid showed a 90% reduction in luciferase activity compared with control mimic-transfected cells (Fig. 2b).

### Reduced expression of LPP3 is associated with renal fibrosis in ZDF rats

**Fig. 3:**
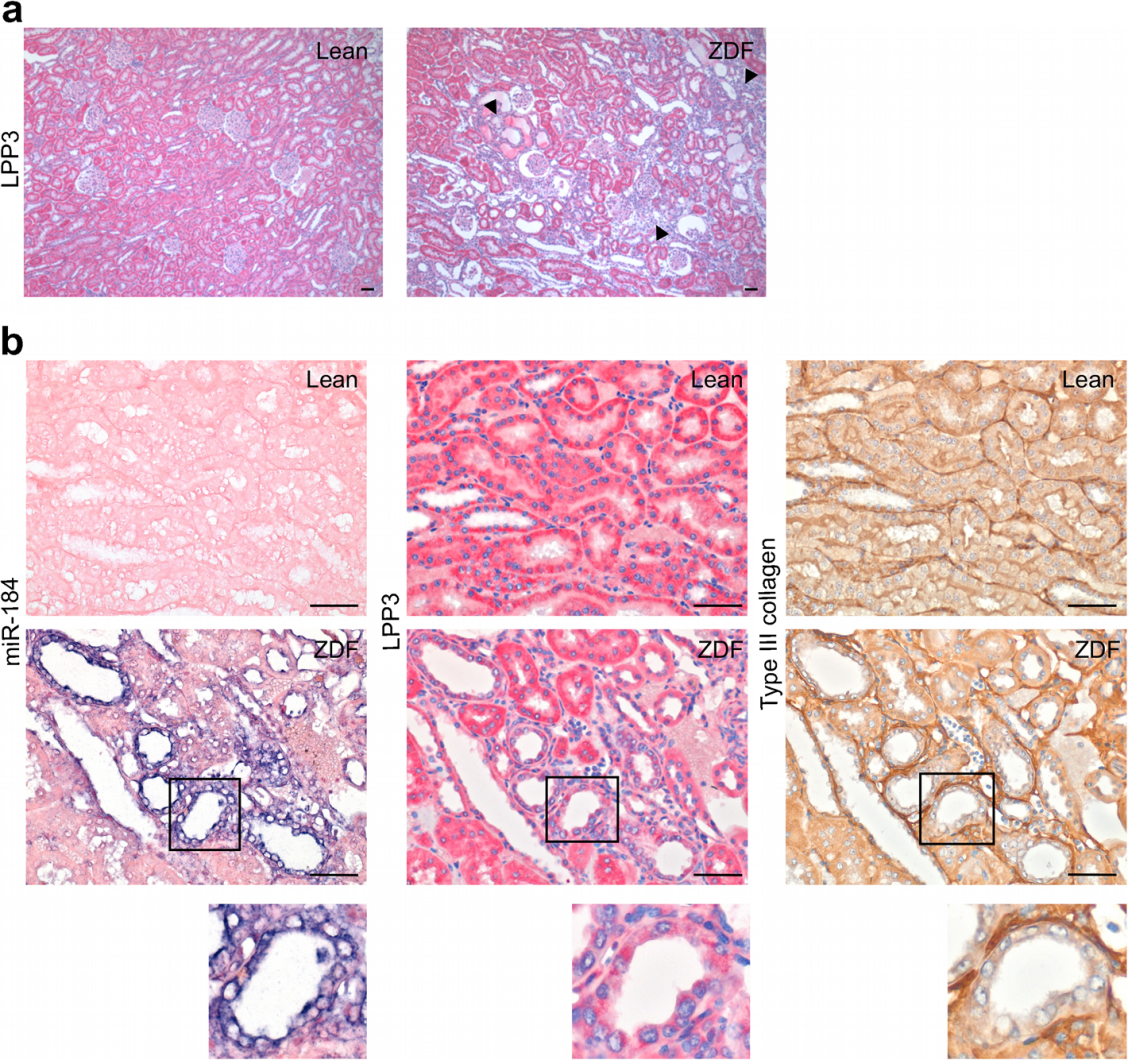
miR-184 upregulation is associated with reduced LPP3 and renal fibrosis in ZDF rats. (**a**) Representative images, at low magnification, of LPP3 expression in lean and ZDF rats. Arrowheads indicate areas of reduced LPP3 staining. (**b**) In situ hybridisation for miR-184 and immunostaining for LPP3 and type III collagen in adjacent kidney sections. Insets show high magnification of a miR184-positive tubule with reduced LPP3 staining and surrounded by interstitial type III collagen deposition. Scale bars, 50 μm

LPP3 is an integral membrane glycoprotein highly expressed in the kidney [21, 22] that catalyses dephosphorylation of lipid phosphates and is engaged in several physiological and pathological processes including fibrosis [23–25]. Immunohistochemistry showed that LPP3 was uniformly expressed throughout the tubule epithelium of lean rats, while in ZDF rats the staining was reduced and limited to focal areas only (Fig. 3a). Serial kidney sections showed that in ZDF rats miR-184 overexpression was associated with reduced LPP3 and accumulation of interstitial type III collagen, used as a marker for fibrosis (Fig. 3b).

### In vitro miR-184 causes LPP3 downregulation accompanied by a profibrotic phenotype of tubule epithelial cells

**Fig. 4:**
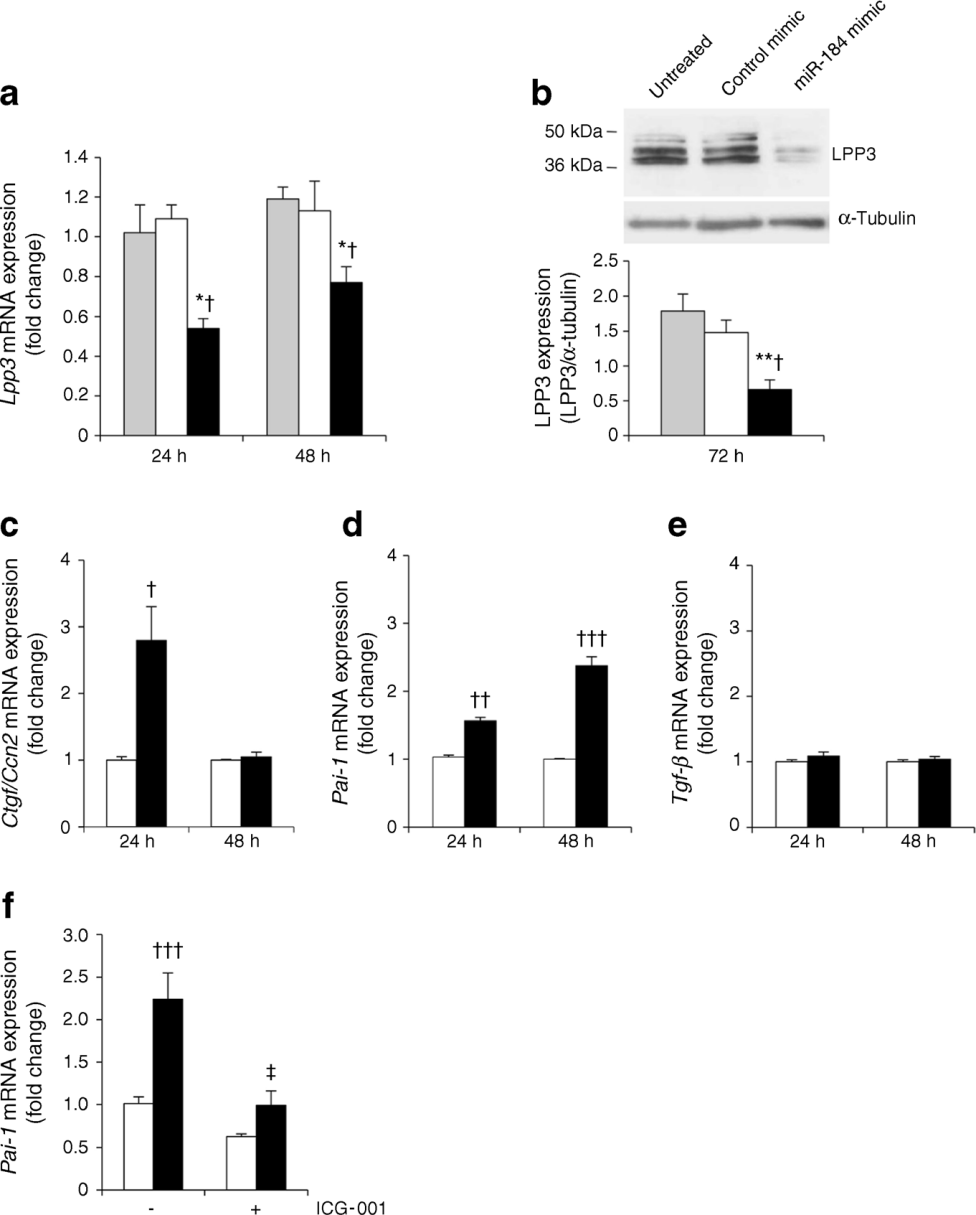
miR-184 downregulates LPP3 and promotes a profibrotic phenotype in proximal tubule cells. (**a**) *Lpp3* mRNA in NRK-52E cells, untreated (grey bars) or transfected with control (white bars) or miR-184 mimic (black bars). (**b**) Representative western blot and densitometric analysis of LPP3. (**c**–**e**) *Ctgf/Ccn2*, *Pai-1* and *Tgf-β* mRNA in cells transfected with control or miR-184 mimic. (**f**) Effect of ICG-001 on *Pai-1* mRNA (48 h) in cells transfected with control or miR-184 mimic. Results normalised to β-actin (**a**, **d**, **f**) or GAPDH (**c**, **e**) are reported as fold change relative to the corresponding control group. *p < 0.05 and **p < 0.01 vs untreated cells; ^†^ *p* < 0.05, ^††^ *p* < 0.01 and ^†††^ *p* < 0.001 vs control mimic-transfected cells at corresponding time; ^‡^ *p* < 0.001 vs miR-184 mimic-transfected cells (*n* = 3 experiments)

We used NRK-52E, a rat proximal tubule cell line, for in vitro mechanistic studies to establish a causal role of miR-184 on LPP3 downregulation and renal fibrosis. Transfection of NRK-52E cells with miR-184 mimic resulted in a significant reduction in *Lpp3* mRNA compared with control mimic-transfected cells, indicating reduced mRNA stability after complementation with miRNA mimic [26] (Fig. 4a). Western blot analysis of LPP3 showed at least two immunoreactive bands with different mobility, ranging from 36 to 50 kDa, which may reflect oligomer formation and/or post-translational protein modifications, such as glycosylation (Fig. 4b) [23, 27]. Consistent with *Lpp3* mRNA downregulation, protein levels were almost halved in the miR-184 mimic-transfected cells (Fig. 4b). Moreover, miR-184 mimic caused a transient increase in *Ctgf*/*Ccn2* mRNA and a sustained upregulation of *Pai-1* mRNA (Fig. 4c,d), while it did not affect *Tgf-β* (Fig. 4e). Increased levels of proinflammatory *Mcp-1* were also observed (ESM Fig. 2). To investigate intracellular mechanisms that link downregulation of LPP3 with upregulation of fibrotic genes we focused on β-catenin-mediated TCF transcriptional activity, which is increased in conditions of LPP3 deficiency [28] and acts as a driver for *Pai-1* transcription [29]. Treatment of cells overexpressing miR-184 with ICG-001, a small-molecule inhibitor of TCF/β-catenin transcription [30], prevented *Pai-1* mRNA upregulation (Fig. 4f), indicating the involvement of the β-catenin–TCF signalling pathway in the profibrotic effect of LPP3 downregulation.

### Albumin is a major regulator of miR-184 in proximal tubule cells

**Fig. 5:**
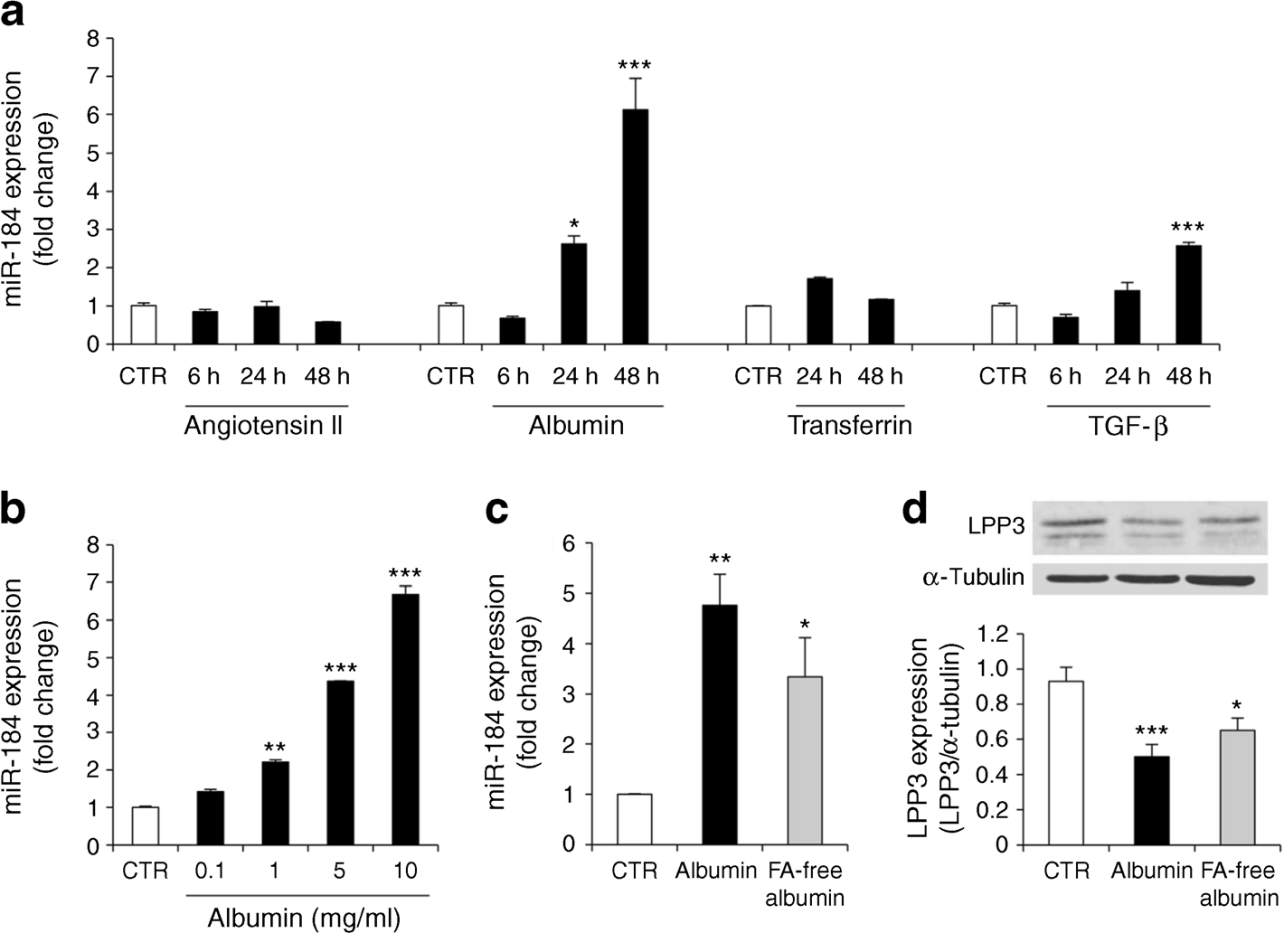
Albumin is the primary regulator of miR-184 in proximal tubule cells. (**a**) miR-184 expression in NRK-52E cells exposed to medium (control, CTR), angiotensin II (10^−7^ mol/l), albumin (10 mg/ml), transferrin (10 mg/ml) or TGF-β (10 ng/ml). (**b**) Dose-dependent effect of albumin on miR-184 expression at 48 h. (**c**) miR-184 expression in cells exposed to medium, albumin and FA-free albumin (10 mg/ml, 48 h). Results normalised to U87 small nucleolar RNA are reported as fold change relative to control cells. (**d**) Representative western blot and densitometric analysis of LPP3 in cells stimulated with albumin or FA-free albumin (72 h). Data are from three (**a**–**c**) or four (**d**) experiments. *p < 0.05, **p < 0.01 and ***p < 0.001 vs control cells

**Fig. 6:**
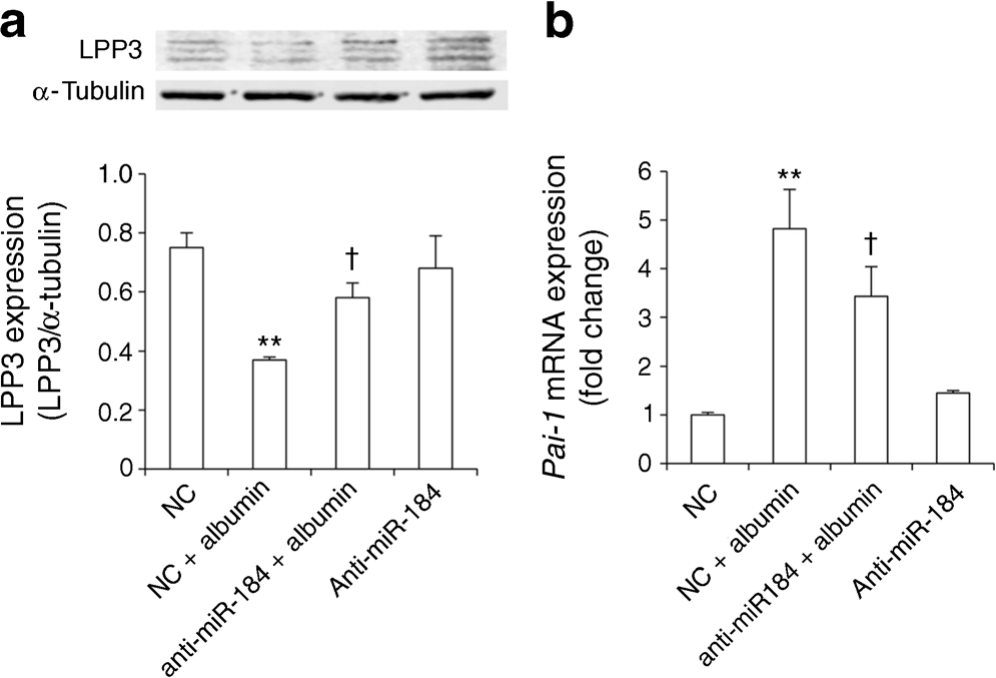
Blockade of miR-184 rescues LPP3 protein and downregulates *Pai-1* mRNA in albumin-treated cells. NRK-52E cells were transfected with anti-miR-184 or miRNA inhibitor negative control (NC), followed by albumin stimulation (10 mg/ml, 72 h). (**a**) Representative western blot and densitometric analysis of LPP3. (**b**) *Pai-1* mRNA expression. Results normalised to β-actin are reported as fold change relative to NC-transfected cells. **p < 0.01 vs NC; ^†^ *p* < 0.05 v NC plus albumin (*n* = 3 experiments)

Angiotensin II, albuminuria and TGF-β contribute to tubulointerstitial injury and fibrosis in chronic renal diseases and diabetic nephropathy [9, 10, 31]. To investigate whether and which of these pathogenic insults could be a trigger for miR-184 expression in renal tubule cells, NRK-52E cells were exposed to the different stimuli for 6–48 h (Fig. 5a). Angiotensin II did not stimulate miR-184. By contrast, albumin was a potent inducer of miR-184, causing a 2.6-fold increase over control cells at 24 h and a 6-fold increase at 48 h. The marked stimulatory effect of albumin was not shared by transferrin, another component of proteinuria known to be toxic to proximal tubule cells [32, 33]. A 2.6-fold increase in miR-184 was induced by TGF-β at 48 h. The increase in miR-184 in cells exposed to albumin was dose dependent, starting with a dose as low as 1 mg/ml (Fig. 5b). Next, we investigated whether FA bound to albumin, rather than albumin itself, was responsible for miR-184 upregulation. Albumin and FA-free albumin (Fig. 5c) caused a 4.8- and 3.5-fold increase in miR-184 expression compared with the control, respectively, suggesting a partial contribution of FA to albumin-induced miR-184 upregulation. Albumin-induced miR-184 expression translated into a 47% reduction in LPP3, compared with control cells (Fig. 5d); a 32% reduction was observed after FA-free albumin. To prove that decrease in LPP3 in response to albumin was dependent on miR-184 upregulation, cells were transfected with anti-miR-184 or with miRNA inhibitor negative control, before albumin incubation. Compared with negative control treatment, anti-miR-184 prevented albumin-induced LPP3 downregulation (Fig. 6a) and limited *Pai-1* overexpression (Fig. 6b), indicating that albumin exerted a profibrotic effect through miR-184/LPP3.

### miR-184 is epigenetically regulated in proximal tubule epithelial cells

**Fig. 7:**
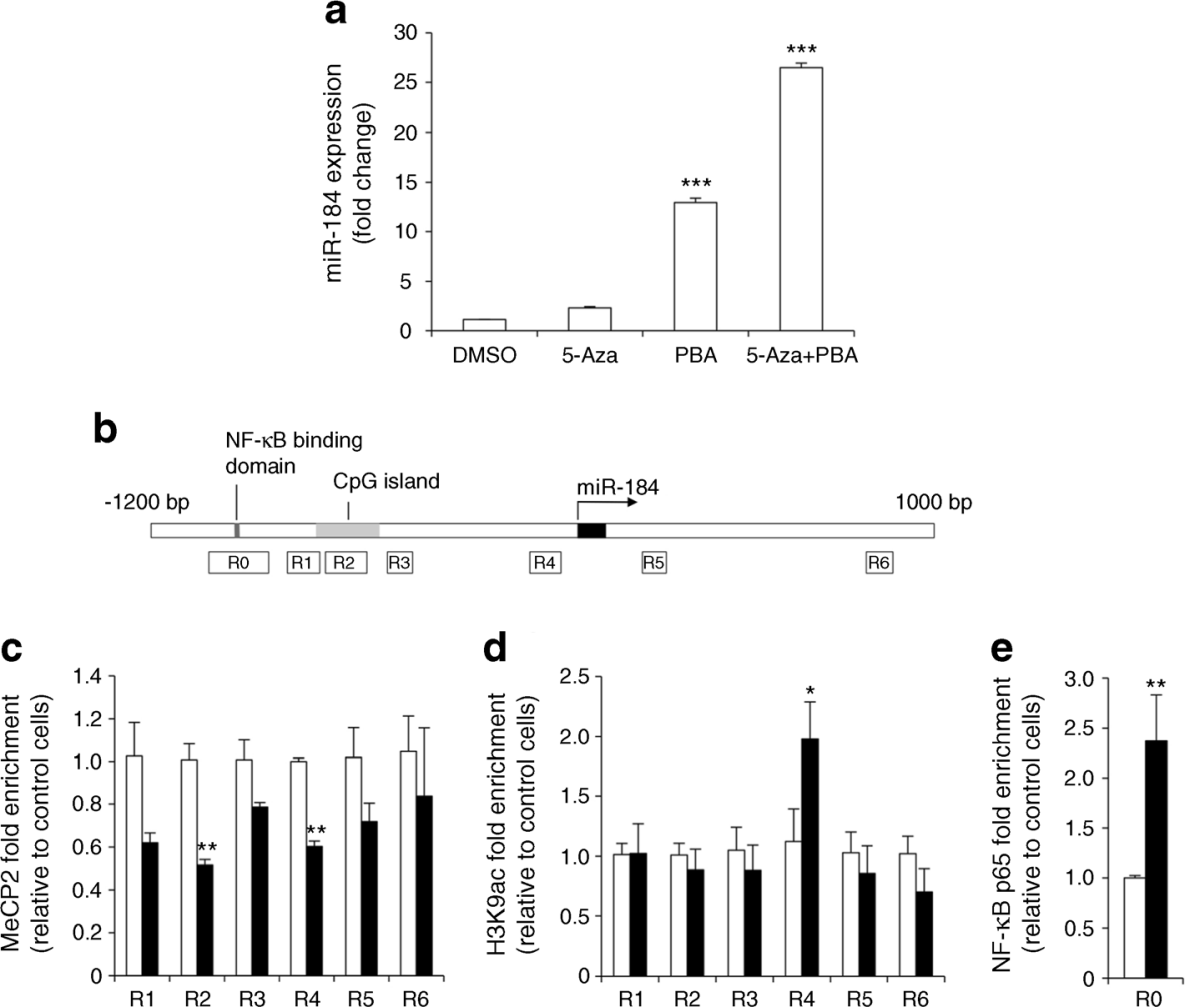
Epigenetic mechanisms involved in albumin-induced miR-184 expression in proximal tubule cells. (**a**) miR-184 expression in NRK-52E cells incubated with vehicle (DMSO), 5-aza-2′-deoxycytidine (5-Aza, 3 μmol/l), phenylbutyric acid (PBA, 3 mmol/l) or their combination. Results normalised to U87 small nucleolar RNA are reported as fold change relative to DMSO-treated cells. *** *p* < 0.001 vs DMSO (*n* = 3 experiments). (**b**) Schematic representation of genomic portion surrounding the miR-184 gene. NF-κB binding domain, putative CpG island and transcription site of miR-184 are indicated. The genomic regions, amplified by ChIP primers, are numbered and shown as rectangles. (**c**–**e**) MeCP2 (**c**), H3K9ac (**d**) and NF-κB p65 (**e**) ChIP in cells without (controls, white bars) or with albumin (10 mg/ml, 48 h, black bars). Input DNA and immunoprecipitated DNA samples were subjected to qPCR using primers spanning the genomic region proximal to the miR-184 gene (R1–R6) for MeCP2 and H3K9ac ChIPs, and primers surrounding the NF-κB binding site (R0) for p65 ChIP. Results normalised to input DNA are expressed as fold enrichment relative to control cells. *p < 0.05 and **p < 0.01 vs control cells (*n* = 3 experiments)

To assess whether epigenetic mechanisms were responsible for miR-184 upregulation, we first exposed NRK-52E cells to the DNA-demethylating agent 5-aza-2′-deoxycytidine, the histone deacetylase inhibitor 4-phenylbutyric acid or their combination. Treating NRK-52E cells with 5-aza-2′-deoxycytidine caused a non-significant increase in miR-184 expression (Fig. 7a). By contrast, 4-phenylbutyric acid significantly upregulated miR-184 expression, which was further enhanced by the combined treatment. This suggests the synergistic effect of DNA demethylation and histone acetylation on chromatin, switching from a silent compact state to an active relaxed state to regulate miR-184 transcription [34, 35]. Next, through computational analysis of the rat genomic region [36] surrounding the miR-184 gene, we identified a putative CpG island located 731 bp upstream of the transcription site of miR-184 (Fig. 7b), suitable for binding methyl-CpG binding proteins, such as MeCP2 and MBD1, known to act as transcriptional repressors [15]. Methyl-CpG binding proteins, together with chromatin histone modifications, are involved in the regulation of miR-184 in mouse brain cells [37–39] and human T cells [39]. We performed ChIP in NRK-52E cells stimulated with or without albumin for 48 h, the time of maximal miR-184 induction, followed by qPCR using primers spanning the CpG island (regions [R]1–3), the regions close to the miR-184 gene (R4–5), and a downstream region (R6) (Fig. 7b). In albumin-treated cells, ChIP-qPCR using MeCP2 antibody revealed a reduction in MeCP2 binding to the genomic region surrounding miR-184 (R1–6), which reached statistical significance within the CpG island (R2) and in the region upstream of the miR-184 transcription site (R4) (Fig. 7c). In contrast, ChIP-qPCR using MBD1 antibody showed a steady but non-significant reduction in MBD1 binding to the analysed genomic regions (ESM Fig. 3a). In addition, we analysed two chromatin markers associated with actively transcribed genes: H3K9ac and H3K4me3. In albumin-stimulated cells, H3K9 acetylation was enriched only in the genomic region upstream of the miR-184 transcription site (R4) (Fig. 7d), whereas H3K4me3 did not show significant enrichment (ESM Fig. 3b). Starting from the evidence of the presence of an NF-κB binding domain 958 bp upstream of the transcriptional site of miR-184 (Fig. 7b), we performed ChIP assay for p65, the NF-κB subunit primarily responsible for transcriptional activation of target genes, and demonstrated that NF-κB was recruited to the miR-184 promoter in albumin-treated cells compared with control cells (Fig. 7e).

### Limiting albuminuria with an ACE inhibitor reduces renal fibrosis in ZDF rats associated with miR-184/LPP3 modulation

**Fig. 8:**
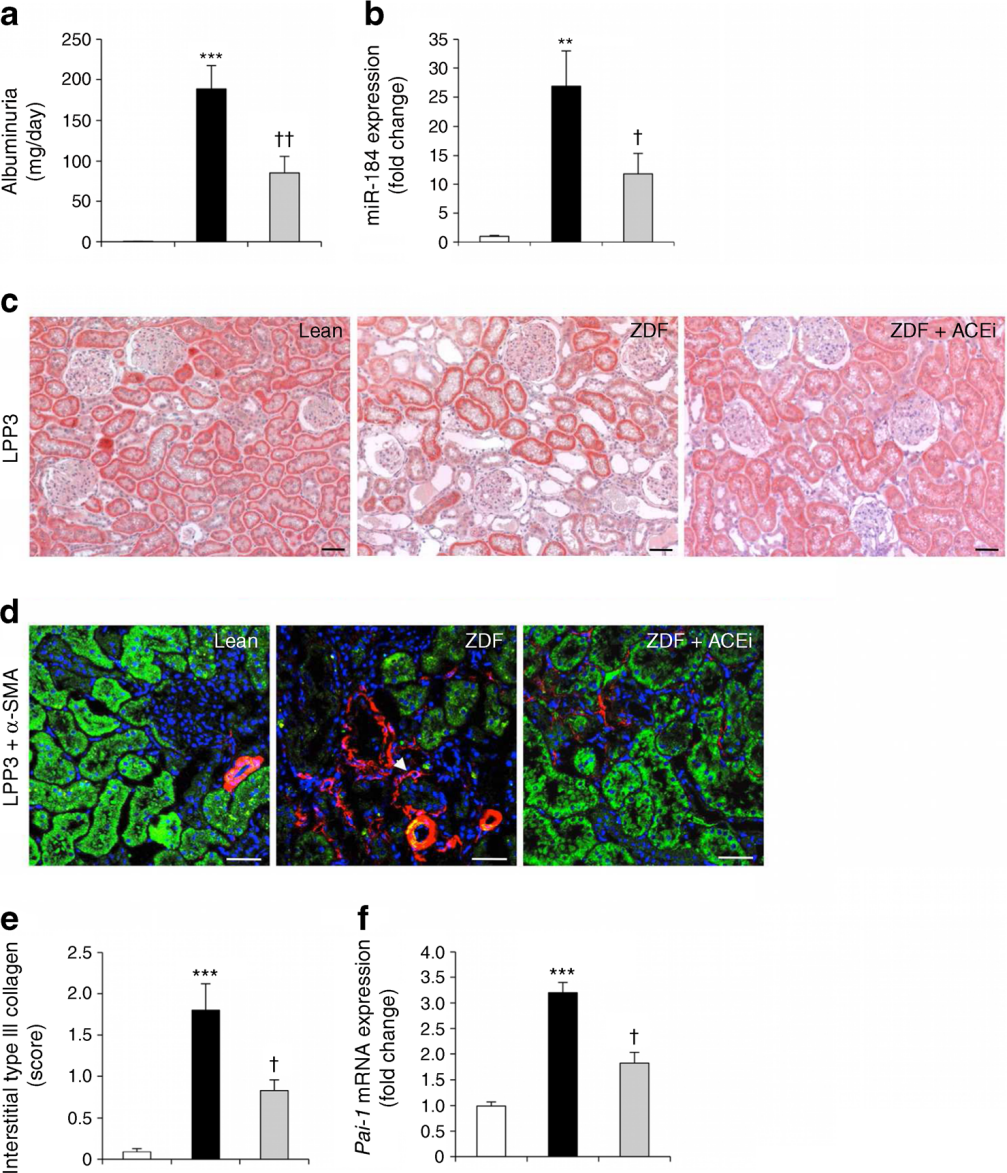
ACE inhibition limits albuminuria in association with modulation of miR-184/LPP3 and amelioration of fibrosis*.* (**a**, **b**) Albuminuria (**a**) and renal miR-184 expression (**b**) measured in lean rats (white bars) and untreated (black bars) or ramipril-treated (grey bars) ZDF rats (*n* = 5/group). Results normalised to U87 small nucleolar RNA are reported as fold change relative to lean rats. (**c**, **d**) Representative images of LPP3 staining (**c**) and double staining for LPP3 (green) and α-SMA (red) (**d**) in kidneys from lean rats, untreated ZDF rats and ZDF rats treated with ramipril (an ACE inhibitor [ACEi]). Scale bar, 50 μm. (**e**, **f**) Quantification of interstitial type III collagen staining (**e**) and renal *Pai-1* mRNA expression (**f**) in lean rats (white bars) and untreated (black bars) or ramipril-treated (grey bars) ZDF rats (*n* = 5/group). Results normalised to β-actin are reported as fold change relative to lean rats. **p < 0.01 and ***p < 0.001 vs lean rats; ^†^ *p* < 0.05 and ^††^ *p* < 0.01 vs untreated ZDF rats

Based on the in vitro data showing the stimulating effect of albumin on miR-184, we moved back to the animal model and investigated whether lowering albuminuria with an ACE inhibitor could limit miR-184 upregulation and consequent fibrosis. Ramipril treatment of ZDF rats with established disease [19], decreased albuminuria by 55% (Fig. 8a) and ameliorated renal function (ESM Table 3) compared with untreated ZDF rats. This was accompanied by reduction in renal miR-184 and preservation of LPP3 in the tubular epithelium (Fig. 8b,c), along with attenuation of fibrosis, as indicated by reduced expression of α-SMA (Fig. 8d), type III collagen (Fig. 8e) and *Pai-1* mRNA (Fig. 8f).

## Discussion

Renal fibrosis is the final common pathway of any form of progressive kidney disease. For many years we have been exploring the molecular mechanisms underlying the development of renal fibrosis and found that dysregulation of the miR-324-3p/Prep complex contributed to the fibrotic process in a model of spontaneous progressive nephropathy [20]. Moving to diabetic nephropathy, here we demonstrated that miR-184 was the most upregulated miRNA in the kidneys of ZDF rats at an advanced phase of the disease. Among mediators of fibrosis, albumin was the most potent stimulus of miR-184, consistent with the putative role of albuminuria in exacerbating disease progression in diabetic nephropathy [40].

Little information is available regarding miR-184 expression in the kidney. Renal miRNA profiling of young and old rodents revealed upregulated miR-184 in old kidneys [41, 42], suggesting that epigenetic regulation of renal ageing likely occurs through inhibition of miR-184 targeted genes encoding antioxidant-, ECM-degrading- and longevity-related proteins [41]. In 8-month-old ZDF rats, miR-184 focally localised in areas of damaged proximal tubules. Tubulointerstitial lesions with deposition of ECM proteins, tubular dilation and atrophy are common findings in progressive chronic kidney diseases and diabetic nephropathy, culminating in renal fibrosis. Here, we suggest a link between abnormal tubular miR-184 and tubulointerstitial fibrosis in the diabetic kidneys through inhibition of the target LPP3, which plays a key role in regulating biosynthesis of lipid phosphates involved in multiple organ fibrosis as well as in cell signal transduction [23–25]. Loss of LPP3 leads to changes in bioactive lipid profile, such as enhanced levels of lysophosphatidate (LPA) [28]. Increased release of LPA and upregulation of LPA1 receptor in kidneys of mice with unilateral ureteral obstruction is associated with development of tubulointerstitial fibrosis [43]. In keeping with this, in the kidneys of ZDF rats, dysregulation of the miR-184/LPP3 pathway would possibly result in increased bioactive lipid phosphate availability, which would activate profibrotic signalling with a consequent accumulation of ECM proteins. Using serial kidney sections, we did document increased type III collagen staining in areas surrounding tubules that were positive for miR-184 and exhibited reduced LPP3 staining. These data were corroborated by in vitro experiments demonstrating that tubule cells showing reduced LPP3 after miR-184 overexpression acquired a profibrotic phenotype documented by enhanced *Ctgf*/*Ccn2* and *Pai-1* mRNAs. The increase in *Ctgf*/*Ccn2* mRNA was transient, consistent with previous observations in proximal tubule cells exposed to LPA [43]. Although the present study did not prove a direct link between LPP3 downregulation and miR-184-induced fibrosis, in vitro experiments suggested β-catenin/TCF signalling as one of the intracellular mechanisms through which miR-184/LPP3 dysregulation may induce profibrotic genes. Indeed, besides its known lipid phosphatase activity, LPP3 may regulate β-catenin activation to the extent that loss of LPP3 resulted in a marked increase in β-catenin-mediated TCF transcriptional activity [28]. Notably, a TCF/LEF-binding site is present in the promoter region of *Pai-1* [29], suggesting PAI-1 as a transcriptional target of the Wnt/β-catenin signalling, known to be involved in renal fibrosis in diabetes [44, 45]. Our data showed that in NRK-52E cells with reduced LPP3 after miR-184 mimic transfection, inhibition of β-catenin–TCF signalling by ICG-001 did prevent *Pai-1* mRNA upregulation.

An interesting observation is that while *Pai-1* and *Ctgf*/*Ccn2* mRNA levels increased in miR-184-overexpressing cells, no changes were observed in *Tgf-β* transcripts. On the other hand, the finding that miR-184 was upregulated in TGF-β1-stimulated cells suggests that this miRNA can likely mediate the cytokine profibrotic effects but, unlike other miRNAs [46], does not contribute to TGF-β auto-upregulation, at least in proximal tubule cells.

One key finding of the present study is that albumin is a major trigger for miR-184 in tubule cells. Albuminuria is one of the best clinical indicators of diabetes-induced renal damage and is a predictor of progression to ESRD [1, 47]. Albuminuria stimulates proximal tubule cells to produce inflammatory and fibrogenic substances capable of either attracting mononuclear cells into the renal interstitium or activating resident fibroblasts and epithelial mesenchymal transition programmes, which contribute to development of fibrosis [31, 48, 49]. Our data, showing that albumin-induced miR-184 in cultured tubule cells was associated with reduced LPP3 and abnormal *Pai-1* mRNA (both prevented by miR-184 antagomir treatment), suggest a functional link between miR-184 dysregulation and albumin load-induced renal fibrosis. Unlike albumin, angiotensin II, a critical mediator of proteinuria and fibrosis [10], failed to induce miR-184 expression in NRK-52E cells, known to express angiotensin II type 1 receptors [50], indicating that angiotensin II lacks a direct effect on miR-184. Importantly, in ZDF rats, lowering albuminuria using an ACE inhibitor was associated with reduced miR-184, preservation of tubular LPP3 and amelioration of tubulointerstitial fibrosis.

**Fig. 9:**
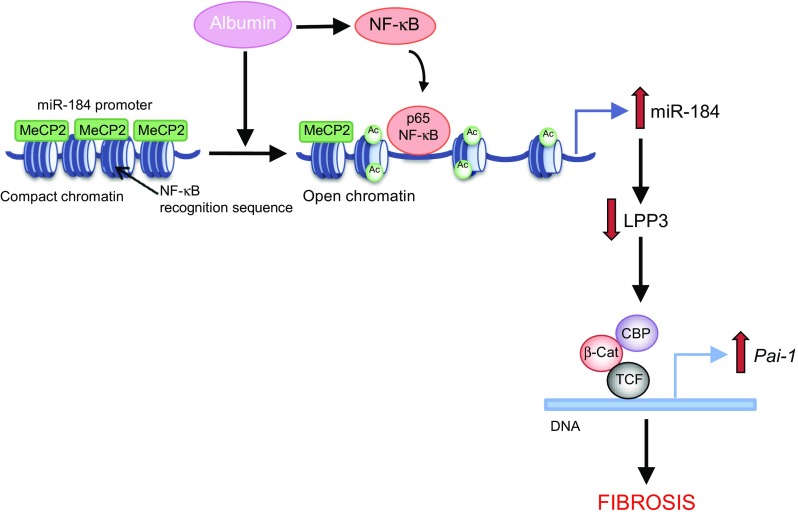
Hypothetical pathways through which albumin overload promotes renal fibrosis via epigenetic regulation of miR-184. Albumin reduces binding of MeCP2 to miR-184 promoter and fosters histone lysine acetylation (Ac), favouring accessibility of NF-κB-p65 to its recognition sequence on the miRNA promoter. This results in miR-184 upregulation and repression of the downstream target LPP3, which in turn upregulates *Pai-1* transcription through the β-catenin (β-Cat)–TCF signalling pathway in a cAMP response-element binding protein (CREB) binding protein (CBP)-dependent fashion

Our data show that albumin has a role in the dysregulation of epigenetic mechanisms like DNA demethylation and histone lysine acetylation, which ultimately lead to miR-184 overexpression in tubule cells. We previously demonstrated that albumin regulates the transcription of proinflammatory and fibrogenic genes through the activation of NF-κB signalling [31]. Importantly, an NF-κB binding site is present in the promoter of miR-184. Our finding that p65 was recruited to miR-184 promoter in albumin-stimulated tubule cells indicates that the chromatin modification events observed in response to albumin make the miR-184 promoter region more accessible to NF-κB, thereby inducing miR-184 transcription (Fig. 9).

In conclusion, this study provides the novel finding that miR-184 is predominantly expressed in the renal tubules of ZDF rats and plays a role in tubulointerstitial fibrosis through downregulation of LPP3. Albuminuria is the main instigator for miR-184 expression in tubule cells under the control of epigenetic mechanisms. These data may offer a new opportunity for targeting miR-184 in association with albuminuria-lowering drugs to achieve fully protective anti-fibrotic effects in diabetic nephropathy.

## Electronic supplementary material

ESM (PDF 1747 kb)

## Abbreviations

α-SMA: Alpha-smooth muscle actin
ChIP: Chromatin immunoprecipitation
CTGF: Connective tissue growth factor
ECM: Extracellular matrix
ESRD: End-stage renal disease
FA: Fatty acids
H3K4me3: Trimethylated histone H3 lysine 4
H3K9ac: Acetylated histone H3 lysine 9
LPA: Lysophosphatidate
LPP3: Lipid phosphate phosphatase 3
MBD1: Methyl-binding domain 1
MCP-1: Monocyte chemoattractant protein-1
MeCP2: Methylcytosine-binding protein 2
miRNA: MicroRNA
PAI-1: Plasminogen activator inhibitor-1
qPCR: Quantitative PCR
qRT-PCR: Quantitative RT-PCR
TCF: T cell factor
UTR: Untranslated region
ZDF rats: Zucker diabetic fatty rats

## References

[1] Remuzzi G, Schieppati A, Ruggenenti P (2002). Clinical practice. Nephropathy in patients with type 2 diabetes. N Engl J Med.

[2] Tuttle KR, Bakris GL, Bilous RW (2014). Diabetic kidney disease: a report from an ADA consensus conference. Am J Kidney Dis.

[3] Najafian B, Alpers CE, Fogo AB (2011). Pathology of human diabetic nephropathy. Contrib Nephrol.

[4] Qian Y, Feldman E, Pennathur S, Kretzler M, Brosius FC (2008). From fibrosis to sclerosis: mechanisms of glomerulosclerosis in diabetic nephropathy. Diabetes.

[5] Hu C, Sun L, Xiao L (2015). Insights into the mechanisms involved in the expression and regulation of extracellular matrix proteins in diabetic nephropathy. Curr Med Chem.

[6] Navarro-Gonzalez JF, Mora-Fernandez C, Muros de Fuentes M, Garcia-Perez J (2011). Inflammatory molecules and pathways in the pathogenesis of diabetic nephropathy. Nat Rev Nephrol.

[7] Loeffler I, Wolf G (2015). Epithelial-to-mesenchymal transition in diabetic nephropathy: fact or fiction?. Cell.

[8] Riser BL, Najmabadi F, Perbal B (2010). CCN3/CCN2 regulation and the fibrosis of diabetic renal disease. J Cell Commun Signal.

[9] Arora MK, Singh UK (2013). Molecular mechanisms in the pathogenesis of diabetic nephropathy: an update. Vasc Pharmacol.

[10] Macconi D, Remuzzi G, Benigni A (2014). Key fibrogenic mediators: old players. Renin-angiotensin system. Kidney Int Suppl (2011).

[11] Zoja C, Locatelli M, Corna D (2016). Therapy with a selective cannabinoid receptor type 2 agonist limits albuminuria and renal injury in mice with type 2 diabetic nephropathy. Nephron.

[12] Kantharidis P, Wang B, Carew RM, Lan HY (2011). Diabetes complications: the microRNA perspective. Diabetes.

[13] Trionfini P, Benigni A (2017) MicroRNAs as master regulators of glomerular function in health and disease. J Am Soc Nephrol doi: 10.1681/ASN.2016101117 10.1681/ASN.2016101117 PMC5461805 28232619

[14] Kato M, Natarajan R (2015). MicroRNAs in diabetic nephropathy: functions, biomarkers, and therapeutic targets. Ann N Y Acad Sci.

[15] Kato M, Natarajan R (2014). Diabetic nephropathy—emerging epigenetic mechanisms. Nat Rev Nephrol.

[16] McClelland A, Hagiwara S, Kantharidis P (2014). Where are we in diabetic nephropathy: microRNAs and biomarkers?. Curr Opin Nephrol Hypertens.

[17] Rudnicki M, Beckers A, Neuwirt H, Vandesompele J (2015). RNA expression signatures and posttranscriptional regulation in diabetic nephropathy. Nephrol Dial Transplant.

[18] Zoja C, Cattaneo S, Fiordaliso F (2011). Distinct cardiac and renal effects of ETA receptor antagonist and ACE inhibitor in experimental type 2 diabetes. Am J Phys Renal Phys.

[19] Zanchi C, Locatelli M, Benigni A (2013). Renal expression of FGF23 in progressive renal disease of diabetes and the effect of ACE inhibitor. PLoS One.

[20] Macconi D, Tomasoni S, Romagnani P (2012). MicroRNA-324-3p promotes renal fibrosis and is a target of ACE inhibition. J Am Soc Nephrol.

[21] Kai M, Wada I, Imai S, Sakane F, Kanoh H (1997). Cloning and characterization of two human isozymes of Mg^2+^-independent phosphatidic acid phosphatase. J Biol Chem.

[22] Barila D, Plateroti M, Nobili F (1996). The Dri 42 gene, whose expression is up-regulated during epithelial differentiation, encodes a novel endoplasmic reticulum resident transmembrane protein. J Biol Chem.

[23] Sciorra VA, Morris AJ (1999). Sequential actions of phospholipase D and phosphatidic acid phosphohydrolase 2b generate diglyceride in mammalian cells. Mol Biol Cell.

[24] Brindley DN, Pilquil C (2009). Lipid phosphate phosphatases and signaling. J Lipid Res.

[25] Pyne NJ, Dubois G, Pyne S (2013). Role of sphingosine 1-phosphate and lysophosphatidic acid in fibrosis. Biochim Biophys Acta.

[26] Huntzinger E, Izaurralde E (2011). Gene silencing by microRNAs: contributions of translational repression and mRNA decay. Nat Rev Genet.

[27] Long JS, Pyne NJ, Pyne S (2008). Lipid phosphate phosphatases form homo- and hetero-oligomers: catalytic competency, subcellular distribution and function. Biochem J.

[28] Escalante-Alcalde D, Hernandez L, Le Stunff H (2003). The lipid phosphatase LPP3 regulates extra-embryonic vasculogenesis and axis patterning. Development.

[29] He W, Tan R, Dai C (2010). Plasminogen activator inhibitor-1 is a transcriptional target of the canonical pathway of Wnt/β-catenin signaling. J Biol Chem.

[30] Eguchi M, Nguyen C, Lee SC, Kahn M (2005). ICG-001, a novel small molecule regulator of TCF/beta-catenin transcription. Med Chem.

[31] Abbate M, Macconi D, Remuzzi G, Zoja C, Alpern RJ, Caplan MJ, Moe OW (2013). Role of proteinuria in the progression of renal disease. Seldin and Giebisch’s the kidney – physiology and pathophysiology.

[32] Tang S, Leung JCK, Tsang AWL, Lan HY, Chan TM, Lai KN (2002). Tranferrin up-regulates chemokine synthesis by human proximal tubular epithelial cells: implication on mechanism of tubuloglomerular communication in glomerulopathic proteinuria. Kidney Int.

[33] Zoja C, Morigi M, Figliuzzi M (1995). Proximal tubular cell synthesis and secretion of endothelin-1 on challenge with albumin and other proteins. Am J Kidney Dis.

[34] Chiurazzi P, Pomponi MG, Pietrobono R, Bakker CE, Neri G, Oostra BA (1999). Synergistic effect of histone hyperacetylation and DNA demethylation in the reactivation of the FMR1 gene. Hum Mol Genet.

[35] Wischnewski F, Pantel K, Schwarzenbach H (2006). Promoter demethylation and histone acetylation mediate gene expression of *MAGE-A1*, *-A2*, *-A3*, and *-A12* in human cancer cells. Mol Cancer Res.

[36] Li LC, Dahiya R (2002). MethPrimer: designing primers for methylation PCRs. Bioinformatics.

[37] Nomura T, Kimura M, Horii T (2008). MeCP2-dependent repression of an imprinted miR-184 released by depolarization. Hum Mol Genet.

[38] Liu C, Teng ZQ, Santistevan NJ (2010). Epigenetic regulation of miR-184 by MBD1 governs neural stem cell proliferation and differentiation. Cell Stem Cell.

[39] Weitzel RP, Lesniewski ML, Greco NJ, Laughlin MJ (2011). Reduced methyl-CpG protein binding contributing to miR-184 expression in umbilical cord blood CD4+ T cells. Leukemia.

[40] Porrini E, Ruggenenti P, Mogensen CE (2015). Non-proteinuric pathways in loss of renal function in patients with type 2 diabetes. Lancet Diabetes Endocrinol.

[41] Bai XY, Ma Y, Ding R, Fu B, Shi S, Chen XM (2011). miR-335 and miR-34a promote renal senescence by suppressing mitochondrial antioxidative enzymes. J Am Soc Nephrol.

[42] Liu X, Fu B, Chen D (2015). miR-184 and miR-150 promote renal glomerular mesangial cell aging by targeting Rab1a and Rab31. Exp Cell Res.

[43] Pradere JP, Klein J, Gres S (2007). LPA1 receptor activation promotes renal interstitial fibrosis. J Am Soc Nephrol.

[44] Xiao L, Wang M, Yang S, Liu F, Sun L (2013). A glimpse of the pathogenetic mechanisms of Wnt/β-catenin signaling in diabetic nephropathy. Biomed Res Int.

[45] Tan RJ, Zhou D, Zhou L, Liu Y (2014). Wnt/beta-catenin signaling and kidney fibrosis. Kidney Int Suppl (2011).

[46] Kato M, Arce L, Wang M, Putta S, Lanting L, Natarajan R (2011). A microRNA circuit mediates transforming growth factor-beta1 autoregulation in renal glomerular mesangial cells. Kidney Int.

[47] Ruggenenti P, Cravedi P, Remuzzi G (2010). The RAAS in the pathogenesis and treatment of diabetic nephropathy. Nat Rev Nephrol.

[48] Zoja C, Abbate M, Remuzzi G (2015). Progression of renal injury toward interstitial inflammation and glomerular sclerosis is dependent on abnormal protein filtration. Nephrol Dial Transplant.

[49] Slyne J, Slattery C, McMorrow T, Ryan MP (2015). New developments concerning the proximal tubule in diabetic nephropathy: in vitro models and mechanisms. Nephrol Dial Transplant.

[50] Zhou L, Xue H, Yuan P (2010). Angiotensin AT1 receptor activation mediates high glucose-induced epithelial-mesenchymal transition in renal proximal tubular cells. Clin Exp Pharmacol Physiol.

