# Supplementary material for: MicroRNA-184 is a downstream effector of albuminuria driving renal fibrosis in rats with diabetic nephropathy
Source: Diabetologia. 2017 Mar 31;60(6):1114–25. doi: 10.1007/s00125-017-4248-9 (PMC5423990; doi:10.1007/s00125-017-4248-9)

ESM Methods

Experimental animals Procedures involving animals were carried out in accordance with institutional guidelines, which were in compliance with national (D.L.n.26, March 4, 2014), and international laws and policies (directive 2010/63/EU on the protection of animals used for scientific purposes) and were approved by the Institutional Animal Care and Use Committees of Mario Negri Institute. Male ZDF rats (ZDF/Gmi-*fa/fa*) and non-diabetic lean rats (ZDF/Gmi-*fa*/+) (Charles River Laboratories Italia S.r.l., Calco, Italy) were kept in specific pathogen-free facility with constant-temperature on a 12 h light-dark cycle with free access to water. ZDF rats were fed Purina 5008 rat chow to accelerate the onset of diabetes. For the first series of experiments two groups of ZDF and lean rats (n=5/group) at 8 months of age were euthanised through CO_2 inhalation and their kidneys were collected and processed for morphological analyses, miRNA profiling, in situ hybridisation and immunohistochemistry experiments. Before sacrifice rats were housed in metabolic cages for 24-hour urine collection for albuminuria assessment. Blood samples were collected for glucose level measurement. In a subsequent study, additional ZDF rats at 4 months of age -when they already developed albuminuria and signs of renal injury [1, 2], -were randomised to receive daily the ACE inhibitor ramipril (1 mg/kg in the drinking water) or vehicle (water) until 8 months (n=5/group). Five lean rats were used as controls. When rats were killed, albuminuria and creatinine clearance were determined. Kidneys were harvested and processed for miR-184, *Pai-1* mRNA expression and for immunohistochemistry. Blood glucose, albuminuria, glomerulosclerosis and tubulointerstitial damage were determined as described [1, 2]. Tubular changes were estimated by scores [2]. Serum and urinary creatinine were measured by Cobas Mira plus autoanalyzer (Roche Diagnostic System, Basel, Switzerland).

MicroRNA expression profiling Total RNA prepared from frozen kidney tissue by miRNeasy isolation kit (Qiagen, Milan, Italy) was reverse-transcribed using the Megaplex RT stem-loop primer pool (Life Technologies, Carlsbad, CA, USA). The cDNA was pre-amplified using a 12-cycle PCR reaction with a miRNA-specific forward primer and universal reverse primer. The miRNA profile was generated using the TaqMan Rodent miRNA specific hydrolysis probes and primers on 7900 HT Fast Real Time PCR system (Life Technologies). Raw miRNA expression data were filtered (Ct values > 32 were considered to be below the limit of reliable detection and excluded) and normalized using the improved version of the global mean normalization method in qbase^{PLUS}[3]. To identify differentially expressed miRNAs statistical analysis was performed using R statistical software (https://cran.r-project.org/) by two nonparametric tests: the Mann-Whitney

test and Rank Products algorithm [4] (p<0.05) and considering only the values with a fold change greater than 2.

Quantitative reverse transcription (qRT)-PCR For miR-184 analysis, RNA enriched for small RNA species was isolated from kidney tissue or NRK-52E cells by mirVana miRNA Isolation Kit (Life Technologies). The cDNA was synthesized with TaqMan microRNA Reverse Transcription primer and MultiScribe Reverse Transcriptase (Life Technologies) and analysed by TaqMan miRNA Assay (ID 000485; Life Technologies) and U87 control miRNA Assay (ID 001712; Life Technologies) as endogenous control. For mRNA analysis, total RNA was isolated from kidney tissue or NRK-52E cells using TRIzol reagent and reverse transcribed with the Superscript III first-strand synthesis system (Life Technologies). *Lpp3*, *Pai-1* and *Mcp-1* were assessed using the following TaqMan assays (Life Technologies): Rn00594311_m1, Rn00561717_m1, and Rn00580553_m1, respectively. *β-actin* was used as endogenous control. *Ctgf/Ccn2* and *Tgf-β* were analyzed using the primers listed in ESM Table 1 and Power SYBR Green PCR Master Mix (Life Technologies). *Gapdh* was used as endogenous control. Amplifications were carried out on ABI ViiA 7 Real-Time PCR system (Life Technologies). Relative quantities were calculated by the $2^{-\Delta\Delta Ct}$ method. Data were presented as fold change relative to the corresponding control group.

***In situ* hybridisation and immunohistochemistry** Hybridisation for miR-184 was performed on 3-μm Duboscq-Brasil-fixed and paraffin-embedded kidney sections. Sections were treated with proteinase K (15 μg/ml; Sigma-Aldrich, St. Louis, MO, USA) for 10 min at 37°C. Hybridisation (1 h at 60°C) was performed using miRCURY microRNA ISH Buffer and the specific double-digoxigenin-labelled LNA miRCURY probe (80 nmol/l; Exiqon, Vedbaek, Denmark). Sections were incubated with alkaline phosphatase-conjugated anti-DIG Fab fragments (1:800; Roche, Mannheim, Germany) and NBT/BCIP substrate (Roche), and counterstained with nuclear Fast Red (Bio-Optica, Milan, Italy). LNA U6 snRNA probe (1 nmol/l; Exiqon) (ESM Fig. 1b) and scramble probe (80 nmol/l; Exiqon) were used as positive and negative controls, respectively.

Immunohistochemical analyses were performed on paraffin-embedded kidney serial sections. For aquaporin1 and Tamm-Horsfall protein stainings, sections were incubated with Peroxidazed1 (Biocare Medical, Concord, CA, USA) to quench endogenous peroxidases after antigen retrieval in DIVA decloaker buffer (Biocare Medical). After blocking with Rodent Block R (Biocare Medical), sections were incubated with rabbit anti-aquaporin1 (1:100; AQP1 H-55, sc-20810, Santa Cruz Biotechnology, Santa Cruz, CA, USA), or rabbit anti-Tamm-Horsfall protein (1:100; THP H-135, sc-20631, Santa Cruz Biotechnology) primary antibodies, followed by rabbit-on-rodent polymer

(Biocare Medical) and diaminobenzidine (Biocare Medical) substrate solution. Slides were finally counterstained with Meyer's hematoxylin. For LPP3 and type III collagen stainings sections, after antigen retrieval in citrate buffer, were incubated with rabbit anti-LPP3 antibody (1:200; orb6643, Biorbyt, Cambridge, UK) or rabbit anti-type III collagen antibody (1:100; AB757P, Chemicon, Temecula, CA, USA). Then, biotinylated secondary antibody was applied, followed by streptavidin-alkaline phosphatase complex (Roche Diagnostics, Basel, Switzerland) for LPP3, and avidin-biotin complex (ABC, Vector Lab, Burlingame, CA, USA) for type III collagen. Stainings were visualized using Fast Red chromogen and diaminobenzidine, respectively. Sections were counterstained with Harris-type hematoxylin. Kidney sections were observed by light microscopy (ApoTome Axio Imager Z2; Zeiss, Jena, Germany). The score of collagen deposition was determined as described [1, 2]. Double immunofluorescence stainings of LPP3 and α-smooth muscle actin (α-SMA) were performed in 3-μm periodate lysine paraformaldehyde-fixed renal samples incubated with rabbit anti-LPP3 primary antibody (1:100; Biorbyt), followed by FITC-conjugated secondary antibody (1:25; Jackson ImmunoResearch Laboratories, West Grove, PA, USA), and Cy3-conjugated mouse anti-α-SMA (1:200; C6198, Sigma-Aldrich). Nuclei were counterstained with DAPI and samples were examined by confocal inverted laser microscopy (LSM 510 Meta; Zeiss). Negative controls were obtained by omitting primary antibodies.

Bioinformatic analysis of miR-184 target genes The microRNA body map web tool (http://www.mirnabodymap.org/, accessed 23 April 2014) (TargetScan, miRDB, MicroCosm, PITA, RNA22, DIANA, TarBAse, miRecords) was used along with the miRanda-mirSVR (http://microRNA.org/, accessed 04 April 2014) and the EIMMo miRNA target prediction software (http://mirz.unibas.ch/EIMMo3/, accessed 04 April 2014) [5] to retrieve potential targets of miR-184.

***In vitro* studies**

Luciferase assay The plasmid containing the human *Lpp3*-3'UTR (*Lpp3*-3'UTR) downstream the Firefly luciferase gene was from Origene (PPAP2B NM_003713 3'-UTR clone in pMirTarget, SC216556, Origene, Rockville, MD, USA). AD-293 cells (240085-41, Agilent, Santa Clara, CA, USA; negative for mycoplasma contamination) were seeded in a 24-well plate (80,000 cells/well) the day before transfection. AD-293 were co-transfected with *Lpp3*-3'UTR (100 ng), the internal control reporter vector pRL-TK (10 ng) encoding the Renilla luciferase and the syn-rno-miR-184 mimic (miScript miRNA mimic, 100 nmol/l; Qiagen) or the Silencer Negative Control siRNA (Control mimic, 100 nmol/l; Ambion, Huntingdon, UK) using Lipofectamine 2000 (Life

Technologies). After 48 h, reporter activity was measured with the Dual-Luciferase Reporter Assay System (Promega, Madison, WI, USA). Firefly luciferase activity was normalised to Renilla luciferase activity of the co-transfected pRL-TK vector. Normalised luciferase activity of control mimic-transfected cells was arbitrarily set to 1.

Cell transfection and incubation The rat proximal tubule cell line NRK-52E (DSMZ, Braunschweig, Germany; negative for mycoplasma contamination) was cultured as described [6]. Before the experiments, cells were serum-starved for 16 h in medium with 2% fetal bovine serum. Syn-rno-miR-184 mimic (miR-184 mimic, 100 nmol/l; Qiagen) or Silencer Negative Control siRNA (control mimic, 100 nmol/l; Ambion) were transfected in NRK-52E cells using Lipofectamine 2000 (Life Technologies). Cells were maintained in serum-free medium and collected 24 and 48 h later for *Lpp3*, *Ctgf/Ccn2*, *Pai-1*, *Tgf-β*, *Mcp-1* mRNA, and 72 h later for western blot of LPP3.

ICG-001 (10 μM; Selleckchem, Munich, Germany) was applied for 48 h to NRK-52E cells transfected with control or miR-184 mimic for *Pai-1* mRNA analysis.

To identify triggers of miR-184 expression, angiotensin II (10^{-7} mol/l), human serum albumin (10 mg/ml), holo-transferrin (10 mg/ml) and TGF-β1 (10 ng/ml) (Sigma-Aldrich) were applied to NRK-52E cells for 6, 24 and 48 h in serum-free medium. Medium was replaced every 24 h with fresh stimuli. For dose-response studies, NRK-52E cells were exposed to albumin at 0.1, 1, 5 and 10 mg/ml for 48 h. Fatty acid (FA)-free human serum albumin (10 mg/ml; Sigma-Aldrich) was used to evaluate the contribution of fatty acids. Anti-rno-miR-184 (miScript miRNA inhibitor, 100 nmol/l; Qiagen) or Silencer Negative Control siRNA (100 nmol/l; Ambion) were transfected in NRK-52E cells before albumin stimulation (10 mg/ml, 72 h). LPP3 protein and *Pai-1* mRNA expression were evaluated.

NRK-52E cells treated with the DNA methyltransferase inhibitor 5-Aza-2'-deoxyxytidine (3 μmol/l) and/or the histone deacetylase (HDAC) inhibitor 4-phenylbutyric acid (3 mmol/l; Sigma-Aldrich) [7] were collected after 48 and 72 h for miR-184 expression. Chromatin-modifying drugs were refreshed every 24 h. Chromatin immunoprecipitation (ChIP) analysis was performed in NRK-52E cells incubated in serum-free medium in the presence or absence of albumin (10 mg/ml, 48 h). Albumin was refreshed every 24 h.

Western blot analysis NRK-52E cells were lysed in RIPA Buffer [6] containing 1X Halt protease inhibitor cocktail (Pierce Thermo Scientific, Rockford, IL, USA). Protein concentration was determined using the BCA Protein Assay (Pierce Thermo Scientific). Equal amounts of proteins (30

μg) were resolved on 12% SDS-PAGE, transferred to nitrocellulose membranes and blocked in 5% milk in TBST 1X (20 mmol/l Tris, 140 mmol/l NaCl, pH7.6 plus 0.1% Tween20). The blots were incubated overnight at 4°C with rabbit anti-LPP3 (1:300; Biorbyt) or mouse monoclonal anti-α-tubulin (1:5,000; T9026, Sigma-Aldrich) antibodies, followed by the appropriate IRDye secondary antibodies (LI-COR Biosciences, Lincoln, NE, USA). The membranes were analysed using the Odyssey Fc infrared image system (LI-COR Biosciences). Bands were quantified by ImageJ Software (National Institute of Health, Bethesda, MD, USA) and normalized to α-tubulin levels.

Chromatin immunoprecipitation Chromatin immunoprecipitation (ChIP) analysis was performed following the Pierce Agarose ChIP Kit (Thermo Scientific) instructions. NRK-52E cells were cross-linked with 1% formaldehyde. Chromatin samples were extracted and digested with Micrococcal nuclease (0.5 U) at 37°C for 15 min. A small fraction of the samples was saved as input DNA sample. Immunoprecipitations were performed overnight at 4°C with the following rabbit antibodies against: methylcytosine-binding protein 2 (MeCP2) (5 μg; ab2828, Abcam), methyl-binding domain 1 (MBD-1) (5 μg; sc-10751, Santa Cruz Biotechnology), acetylated histone H3 lysine 9 (H3K9ac) (2 μg; ab4441, Abcam, Cambridge, UK), trimethylated histone H3 lysine 4 (H3K4me3) (2 μg; ab8580, Abcam) and NF-κB p65 (5 μg; SC-372X, Santa Cruz Biotechnology). Anti-RNA polimerase II antibody and normal rabbit IgG (Pierce Agarose ChIP Kit, Thermo Scientific) were used to generate positive and negative controls, respectively. Genomic DNA was purified and amplified by quantitative (q)PCR using a set of primers designed to amplify different portions of the genomic region surrounding the miR-184 gene. Primer sequences are listed in ESM Table 1. Data were analysed by using the $2^{-\Delta\Delta Ct}$ method and normalized with input samples. Results were expressed as fold enrichment over control cells.

References

[1] Zoja C, Cattaneo S, Fiordaliso F et al. (2011) Distinct cardiac and renal effects of ETA receptor antagonist and ACE inhibitor in experimental type 2 diabetes. Am J Physiol Renal Physiol 301:F1114-1123

[2] Zanchi C, Locatelli M, Benigni A et al. (2013) Renal expression of FGF23 in progressive renal disease of diabetes and the effect of ACE inhibitor. PLoS One 8:e70775

[3] D'Haene B, Mestdagh P, Hellemans J et al. (2012) miRNA expression profiling: from reference genes to global mean normalization. Methods Mol Biol 822: 261-272

[4] Breitling R, Armengaud P, Amtmann A, Herzyk P (2004) Rank products: a simple, yet powerful, new method to detect differentially regulated genes in replicated microarray experiments.

FEBS Lett 573:83-92
[5] Gaidatzis D, van Nimwegen E, Hausser J, Zavolan M. (2007) Inference of miRNA targets using evolutionary conservation and pathway analysis. BMC Bioinformatics: 1;8:69
[6] Macconi D, Tomasoni S, Romagnani P et al. (2012) MicroRNA-324-3p promotes renal fibrosis and is a target of ACE inhibition. J Am Soc Nephrol 23: 1496-1505
[7] Saito Y, Liang G, Egger G et al. (2006) Specific activation of microRNA-127 with downregulation of the proto-oncogene BCL6 by chromatin-modifying drugs in human cancer cells. Cancer Cell 9: 435-443

ESM Table 1 Sequences of the primers used for qRT-PCR and ChIP-qPCR

Gene	Forward (5' to 3')	Reverse (5' to 3')	Amplicon length
cDNA primers			
Ctgf/Ccn2	CTAGCTGCCTACCGACTGGAA	CTTAGAACAGGCGCTCCACT	99
Tgf-β	GCTGAACCAAGGAGACGGAAT	AAGAGCAGTGAGCACTGAAGC	51
Mcp-1	CAGATGCAGTTAATGCCCCA	TCTTGCCAGTGAATGAGTAGCAG	51
Gapdh	TCATCCCTGCATCCACTGGT	CTGGGATGACCTTGCCCA	56
ChIP primers			
Region 0 (R0)	TGCACACAGAGGAATATATGAAAAC	TTGAGCCTCAGTTTCCAGGC	169
Region 1 (R1)	ATAAAGTTGATGCCTTCCCCTCA	GACACCATTCCGTTTCCCACA	90
Region 2 (R2)	GCTGTTCCCACTTCTTTCGG	GTGCTCACGTAACCAGCTCA	115
Region 3 (R3)	CAGGGCAGACACATCCAGTC	ATTGGTCCTCCAGAGAGGCT	70
Region 4 (R4)	AGAAGAGCTAGAGGGACCCG	AAGCTGACACCTGGTTTCCC	88
Region 5 (R5)	CGCAGGACTTGAGGAGGAAG	CTCAGGCAGGAAGCTCAGAC	65
Region 6 (R6)	CGCCAATGGACAAATGGTGG	GGCTCGACTTCGTTCGTACA	76

ESM Table 2 List of fold changes of miRNAs differentially expressed in ZDF rats compared with lean rats

miRNA	Fold Change
miR-184	18.09
miR-132-3p	4.31
miR-212-3p	3.01
miR-205	2.72
miR-503-5p	2.72
miR-21-5p	2.57
miR-224-5p	2.47
miR-449a-5p	2.45
miR-708-5p	2.43
miR-155-5p	2.42
miR-802-5p	0.44
miR-489-3p	0.4
miR-375-3p	0.34
miR-592	0.3
miR-10b-3p	0.13

ESM Table 3 Creatinine clearance in lean and ZDF rats

Groups	Creatinine clearance (ml/min/100g)
Lean	0.63 ± 0.01
ZDF + vehicle	0.42 ± 0.05
ZDF + ramipril	0.57 ± 0.13

Data are mean ± SEM (n=5 rats/group). ZDF rats were daily treated from 4 to 8 months of age with vehicle or ramipril (1mg/kg in the drinking water)

ESM Figure 1 (a) miR-184 expression in the glomeruli from a ZDF rat at 8 months of age as evaluated by in situ hybridisation (arrows mark glomerular cells, likely podocytes positive for miR-184). **(b)** In situ hybridisation for LNA U6 snRNA probe as positive control in a ZDF rat kidney section. Scale bars 50 μm

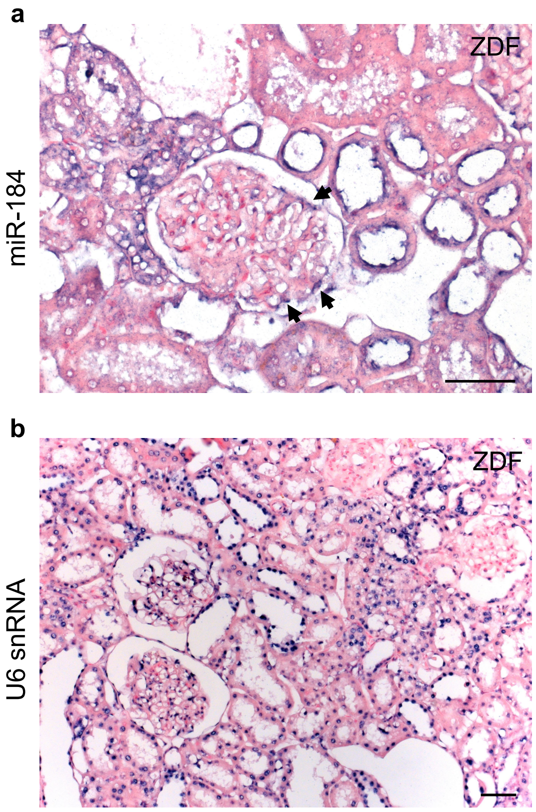

ESM Figure 2 *Mcp-1* mRNA expression in NRK-52E cells transfected with control (white bars) or miR-184 mimic (black bars). Results, normalised to GAPDH, are reported as fold changes relative to control mimic-transfected cells at the corresponding time. Data are mean ± SEM from three experiments. *p<0.01 versus control mimic-transfected cells at the corresponding time

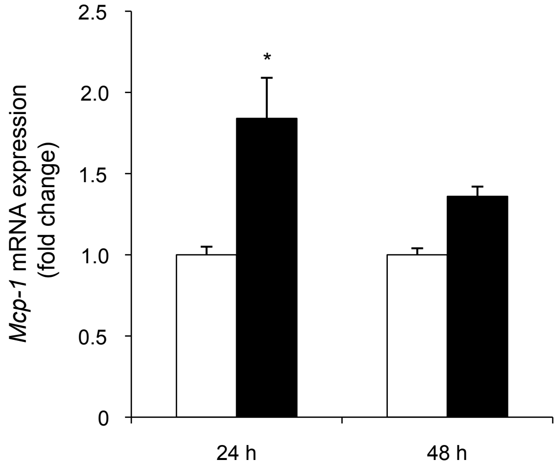

ESM Figure 3 (a) MBD1 and **(b)** H3K4me3 chromatin immunoprecipitations performed in NRK-52E cells untreated (white bars) or stimulated with albumin (10 mg/ml, 48 h) (black bars). Input DNA and immunoprecipitated DNA samples were subjected to qPCR with primers spanning the genomic region around the miR-184 gene. Results, normalized to input DNA, are expressed as fold enrichment relative to control cells. Data are mean ± SEM of three experiments

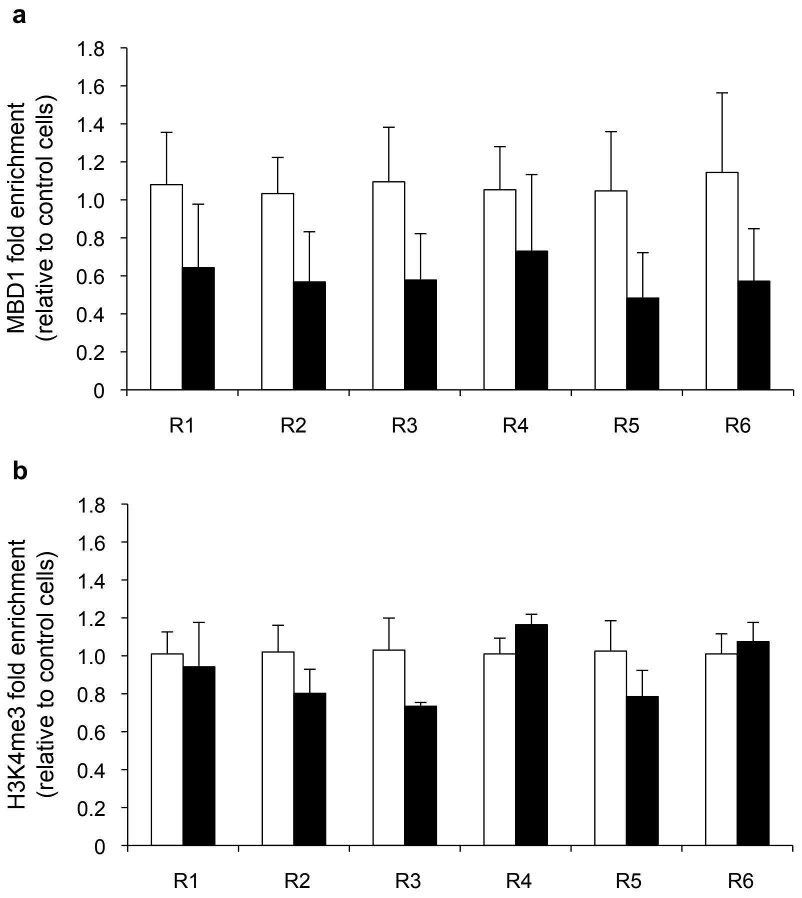

Supplement: Supplementary file 1 — (PDF 1747 kb) [file 125_2017_4248_MOESM1_ESM.pdf]
